# The Warburg Trap: A Novel Therapeutic Approach for Targeting Osteosarcoma

**DOI:** 10.3390/cells13010061

**Published:** 2023-12-27

**Authors:** Joerg Fellenberg, Sarina Losch, Elena Tripel, Burkhard Lehner, Svitlana Melnik

**Affiliations:** Department of Orthopaedics, University Hospital Heidelberg, 69118 Heidelberg, Germany; sarina.losch@med.uni-heidelberg.de (S.L.); elena.tripel@med.uni-heidelberg.de (E.T.); burkhard.lehner@med.uni-heidelberg.de (B.L.); svitlana.melnik@gmail.com (S.M.)

**Keywords:** osteosarcoma, Warburg Trap, chemotherapy, apoptosis, cancer stem cells

## Abstract

Although urgently needed, no significant improvements in osteosarcoma (OS) therapy have been achieved within the last decades. Here, we present a new therapeutic approach based on drug combinations consisting of mitochondrial complex I (MCI) inhibitors and ionophores that induce cancer cell-specific cell death based on a modulation of cellular energy metabolism and intracellular pH (pHi) named the Warburg Trap (WT). The effects of several drug combinations on intracellular pH, cell viability, colony-forming capacity and expression of WNT-target genes were analysed using OS cell lines and primary human osteoblasts (HOB). Tumour take rates and tumour volumes were analysed in vivo using a chicken chorioallantoic membrane assay (CAM). Several WT drug combinations induced the intracellular acidification and apoptotic cell death in OS cells, whereas HOBs tolerated the treatment. A significant inhibition of the colony-forming ability of OS cells and downregulation of WNT-target genes suggest that cancer stem cells (CSCs) are also targeted by the WT approach. In vivo, we observed a significant reduction in the tumour take rates in response to WT drug treatment. Our data suggest that the Warburg Trap is a promising approach for the development of a novel and effective OS therapy to replace or supplement the current OS chemotherapy.

## 1. Introduction

Osteosarcoma (OS) is the most common type of malignant bone cancer predominantly affecting the distal femur or the proximal tibia of children and young adults and accounts for 5% of all cancers among this age group [1]. Current therapy strategies include neoadjuvant and adjuvant chemotherapy applied in most cases of OS as well as surgery for localised OS that, depending on the tumour tissue spread, may result in limb salvage or limb amputation. In rare cases of inoperable tumours, radiotherapy is applied [2]. Even though the five-year survival rate for non-metastatic osteosarcoma is 60–70%, the survival rate for patients with metastatic or recurrent disease is less than 25% [3,4]. In addition, recurrent osteosarcoma frequently develops resistance to chemotherapy. Therefore, even though it is a rare type of malignancy, the lack of progress concerning treatment strategies makes it the second most common cause of death in this age group [5].

At present, the most effective OS treatment strategy consists of chemotherapy protocols based on DNA-damaging cytotoxic drugs and includes a combination of high-dose methotrexate, doxorubicin, and cisplatin (MAP regimen) [6]. Unfortunately, no significant improvements in this therapy approach have been achieved over the last four decades [7,8,9]. For example, the introduction of new biologic agents such as Muramyl tripeptide [10] and the use of additional cytotoxic drugs such as Ifosfamide [11], or Interferon α [12] have not improved the survival of osteosarcoma patients. Also, the latest and largest multinational therapy-optimising study “Euramos-1”, evaluating a multi-drug chemotherapy regimen in over 2000 OS patients, could not contribute to improved survival for osteosarcoma patients [12]. Thus, new therapeutic strategies that replace or complement conventional chemotherapy are urgently needed.

The main problem of all drug-based anti-cancer treatments to date is their high toxicity and the occurrence of side effects in non-cancer cells and tissues. Thus, an effective therapy should target some very specific cancer cell properties that are absent in non-cancer cells. It has been known for a long time that cancer cells, indeed, have such distinct metabolic properties. The first hallmark of cancer metabolism is a preference for anaerobic glycolysis over the oxidative phosphorylation pathway characteristic to normal non-cancer cells—the Warburg effect [13]. As a consequence, cancer cells accumulate lactic acid that needs to be transported across cell membranes through H^+^/lactate monocarboxylic acid transporters (MCT1 and MCT4). Lactate over-production in turn results in a reversed pH gradient, another metabolic feature and the second hallmark of cancer metabolism. The reversed pH gradient then leads to the manifestation of extracellular acidosis and intracellular alkalisation.

Earlier, we demonstrated that these distinct properties of cancer metabolism can be successfully exploited [14]. We formulated a general principle stating that all drug combinations that escalate cancer cells into hyper-acidification and ATP depletion would trigger an irreversible cycle leading to cancer cell death. For this, we combined mitochondrial complex I inhibitors (MCIis) (e.g., Papaverine [15], Metformin [16] or Phenformin) with an H^+^-Ionophore (e.g., Monensin [17], or Drotaverine, an anti-spasmodic drug causing the pHi drop by unknown mechanisms. This combination resulted in a self-enhancing cycle that we named the “Warburg Trap”. We have shown that once a cancer cell entered such a cycle, it used all accessible ATP until complete exhaustion in an attempt to restore the homeostatic pHi. Thus, the simultaneous presence of H^+^-Ionophore and MCIi drugs forced cancer cells into acidosis and apoptosis. In contrary, normal cells—that are known to have lower sensitivity to MCIi [18]—had no restriction to activate the tricarboxylic acid (TCA) cycle to restore and maintain physiological levels of ATP and the pHi. Notably, we have observed no toxicity in corresponding in vitro and in vivo experiments and confirmed the proposed mechanism for cancer cell-specific effects of the Warburg Trap drugs using a mouse tumour xenograft model. So far, however, nothing is known about the suitability of this method for the treatment of osteosarcoma. Therefore, this study aimed to transfer and verify this mechanism in an osteosarcoma environment and to assess the feasibility of the WT approach as a novel treatment option to replace or complement conventional OS chemotherapy.

## 2. Materials and Methods

### 2.1. Cell Origin and Isolation

The following six commercially available human osteosarcoma cell lines were used in this study: HOS (American Tissue Culture Collection, Rockville, MD, USA), HOS 143B (Sigma-Aldrich, Taufkirchen, Germany), CAL-72 (DSMZ, Braunschweig, Germany), MNNG-HOS, SaOS-2, and U2OS (Cell Line Service GmbH, Eppelheim, Germany).

Primary human osteoblasts (HOBs) were isolated from bone chips derived from patients with diagnosed osteoarthritis of the knee undergoing a complete knee replacement surgery. After removal of the cartilaginous areas, bone material was washed several times with PBS (Thermo Fisher Scientific, Karlsruhe, Germany), cut into small fragments of 1–3 mm in size and digested in Dulbecco’s Modified Eagle Medium (DMEM) supplemented with 1 mg/mL Collagenase Type II (Thermo Fisher Scientific) for 2 h at 37 °C under continuous rotation. After digestion, cells were washed twice in PBS and cultured in DMEM (Thermo Fisher Scientific) containing 1 g/L glucose, 10% foetal calf serum (FCS) (Thermo Fisher Scientific), 100 U/mL penicillin/streptomycin (Sigma-Aldrich), and 0.17 mM ascorbic acid 2-phosphate (Sigma-Aldrich). After 24 h, non-adherent cells were carefully removed without disturbing bone fragments. At 80% confluency, cells were passaged, and the bone fragments were discarded.

### 2.2. Cell Culture Conditions and Treatment

All osteosarcoma cell lines were cultured in Dulbecco’s Modified Eagle Medium (DMEM) (Thermo Fisher Scientific) containing 4.5 g/L glucose, 10% FCS (Thermo Fisher Scientific), and 100 U/mL penicillin/streptomycin (Sigma-Aldrich) under standard cell culture conditions at 37 °C and 5% CO_2_ in a humidified atmosphere. HOBs were cultured as described above. The medium was changed twice per week, and cells were passaged at 80% confluency.

Drug treatment was performed in RPMI 1640 medium (Thermo Fisher Scientific) supplemented with 10% FCS (Thermo Fisher Scientific), 20 mM PIPES (Carl Roth, Karlsruhe, Germany), 1 mM sodium–pyruvate (Sigma-Aldrich) and 100 U/mL penicillin/streptomycin (Sigma-Aldrich). The following drugs were used: Metformin, Monensin, Fenofibrate, Drotaverine and Papaverine (all Biomol, Hamburg, Germany). If not otherwise stated, the following drug concentrations were used: Metformin (1 mM), Monensin (5 nM), Fenofibrate (10 µM), Drotaverine (10µM), and Papaverine (10µM). For the preparation of acidic cell culture medium, the pH value of the above-mentioned RPMI medium was adjusted with a PIPES buffer to 6.5. The normal cell culture medium was replaced by acidic medium 24 h before the experiments.

### 2.3. Cell Viability Analysis

Cell viability was quantified using a water-soluble tetrazolium salt (WST-1) assay (Roche Diagnostics, Mannheim, Germany). Cells were plated in 96-well plates (15,000 cells/well) in the desired culture medium 24 h before treatment. After treatment, the medium was replaced by 100 µL WST-1 reagent diluted 1:10 with cell culture medium and incubated for 120 min at 37 °C. Finally, optical absorbance was determined in a plate reader (Autobio-Phomo, Anthos Microsystems, Friesoyte, Germany) at 450 nm with a reference wavelength of 600 nm. Wells without cells were used as blank controls, and their values were subtracted from the values of the experimental samples. All measurements were carried out in duplicates.

### 2.4. Measurement of Intracellular pH

Cells were plated in 96-well plates at a density of 15,000 cells/well and cultured for 24 h at 37 °C and 5% CO_2_ in a humidified atmosphere. After treatment, the medium was replaced by 100 µL staining solution consisting of the pH-sensitive fluorescent dye BCFL (5 µM) (Biomol) diluted in Hanks balanced salt solution (HBSS) (Sigma-Aldrich). Following 30 min incubation at 37 °C, the staining solution was removed, cells were washed twice with HBSS buffer, and fluorescence was quantified at 535 nm using a FluoStar fluorescence microplate reader (BMG LabTech, Ortenberg, Germany). For quantification, standard curves were generated using culture media with pH values ranging from pH 6.0 to pH 8.0 supplemented with the ionophore Nigericin (10 µM) (Sigma-Aldrich) that caused an adjustment of the intracellular pH value to the extracellular pH-value. Based on these standards, pH values of the experimental groups were calculated.

### 2.5. Colony Forming Unit Assay

For drug pre-treatment, 10^5^ cells were plated per well in 12-well plates and incubated for 24 h before the cell culture medium was replaced by an acidic culture medium containing the desired drugs. After drug pre-treatment for 24 or 48 h, cells were trypsinised, counted and replated in 6-well cell culture plates at a density of 2500 cells per well. Colonies that formed within 10 days were fixed for 10 min in ice-cold methanol (Carl Roth GmbH, Karlsruhe, Germany), washed with PBS and stained with Gill’s haematoxylin solution (Santa Cruz, Heidelberg, Germany) for 3 min. After rinsing in tap water for 7 min, colonies were photographed in 12 individual fields of view per well before the number and size of the colonies were quantified using ImageJ software version 1.48v (https://imagej.net/ij/ accessed on 3 July 2023).

### 2.6. Soft Agar Assay

After drug pre-treatment for 24 or 48 h, as described above for the colony-forming unit assay, cells were subjected to the soft agar assay. The assay was carried out in 24-well culture plates that were coated with 300 µL DMEM containing 4.5 g/L glucose, 10% FCS (Thermo Fisher Scientific), 100 U/mL penicillin/streptomycin (Sigma-Aldrich) and 0.5% agarose (Biozym, Hessisch Oldendorf, Germany). Above this base layer, a top layer was poured containing 5000 cells in 500 µL DMEM cell culture medium supplemented with 0.3% agarose pre-heated to 42 °C. After hardening of the agarose layers, 1 mL DMEM culture medium was added to each well. Cells were incubated at 37 °C in a humidified atmosphere for 10 days before colonies (>20 cells) were counted using a microscope. Experiments were performed with six different osteosarcoma cell lines in duplicates.

### 2.7. RNA Extraction, cDNA Synthesis and RT-qPCR

Total RNA was isolated using the RNeasy Mini kit (Qiagen, Hilden, Germany) according to the manufacturer’s instructions. Concentrations and quality of the extracted RNAs were analysed using a NanoDrop ND-1000 spectrophotometer (Peqlab, Erlangen, Germany). First-strand complementary DNA (cDNA) was synthesised from 250 ng of total RNA using a cDNA synthesis kit (Biozym). Reactions were carried out for 1 h at 37 °C in a total volume of 20 µL. After synthesis, cDNA was further diluted 1:5 with 10 mM Tricine buffer (Carl Roth GmbH). Then, 2 µL of each cDNA was subjected to RT-qPCR analysis using primaQuant QPCR master mix (Steinbrenner Laborsysteme, Wiesenbach, Germany). The expression of *RPS13* (ribosomal protein S13) in the corresponding sample was used for normalisation. All primers used for RT-qPCR are listed in Supplementary Table S1.

### 2.8. Detection of Apoptotic Cells

The induction of apoptosis was quantified by NucViev 488 (Biomol) staining that detects the activation of caspase-3, which is a hallmark of apoptosis. Cells were seeded at a density of 20,000 cells per well in black 96-well cell culture plates with a clear bottom. On the next day, the culture medium was replaced by an acidic RPMI culture medium (pH 6.5) with or without the addition of the WT drugs Metformin (1 mM), Monensin (5 nM) and Fenofibrate (10 µM). After treatment for 24 h and 48 h, respectively, cells were stained with NucView 488 (2.5 µM), diluted in Hank′s balanced salt solution (HBSS) (Sigma-Aldrich) for 30 min at 37 °C and photographed using a BZ-X800 microscope (Keyence, Neu-Isenburg, Germany). The quantification of apoptotic cells was carried out by flow cytometric analysis of NucView 488 stained cells using a MacsQuant analyser (Miltenyi Biotec, Bergisch Gladbach, Germany).

### 2.9. Chicken Chorioallantoic Membrane (CAM) Assay

The effects of the Warburg Trap drugs on tumour growth under in vivo conditions were analysed using a CAM assay protocol that we previously established and optimised for the xenotransplantation of osteosarcoma cells [19]. In brief, upon arrival, fertilised chicken eggs obtained from a local hatchery (Geflügelzucht Hockenberger, Eppingen, Germany) were cleaned with dry paper towels and incubated at 37.8 °C at 70% humidity and permanent agitation. After three days of incubation, 3 mL albumen was removed with a syringe before a window was carved into the eggshell and resealed with a Durapore tape (3M, Neuss, Germany). This opening was then used on day 9 for transplanting OS cells (HOS 143B) onto the chorioallantoic membrane. For the transplantation, 1 × 10^6^ cells were resuspended in 20 µL RPMI 1640 medium (pH 6.5) (Thermo Fisher Scientific), with or without drugs, and mixed with an equal volume of Cultrex BME Type 3 (AMS Biotechnology, Frankfurt, Germany), which served as a matrix and nutrient supply. A silicone ring was applied onto the CAM through the window, and the CAM within this ring was gently lacerated to promote subsequent vascularization. After transplantation of 40 µL cell-matrix suspension into the center of the silicone ring, eggs were closed and incubated for further seven days. On day 16, embryos were euthanized using the pentobarbital Narcoren before formed tumours were removed. The tumour take rates were calculated as number of eggs with tumours × 100/number of eggs with vital embryos. Tumour volumes were calculated using the following formula: volume = 4/3 × π × r^3^ (r = 1/2 × √ of diameter 1 × diameter 2) [20]. At least 20 eggs were used for each experimental group.

### 2.10. Lens Culinaris Agglutinin Staining

Detection of chicken cells in paraffin-embedded tumour tissue was carried out by Lens Culinaris Agglutinin staining as described previously [21]. In brief, sections were deparaffinised, rehydrated and blocked with 5% bovine serum albumin (BSA) for 15 min before they were incubated with 5 µg/mL biotinylated Lens Culinaris Agglutinin (Linaris, Dossenheim, Germany) that was diluted in PBS for 30 min at room temperature. After washing in PBS, samples were incubated for 30 min in AB reagent (Avidin D/biotinylated alkaline phosphatase) (Linaris), washed again and stained with the alkaline phosphate substrate ImmPACT Vector Red (Linaris). After counterstaining with Methyl Green (Linaris), sections were photographed using a Keyence BZ-X800 microscope (Keyence).

### 2.11. ALU In Situ Hybridisation

The detection of human cells within the resected paraffin-embedded tumour tissues was performed as described previously [21]. In brief, deparaffinised and rehydrated sections were digested for antigen retrieval with 5 µg/mL proteinase K (Roche Diagnostics, Mannheim, Germany) for 15 min at 37 °C. Prehybridisation was completed at 42 °C for 1 h in a hybridisation buffer consisting of 4 × SSC (saline sodium citrate), 50% deionised formamide, 1 × Denhardt’s solution, 5% dextran sulfate and 100 µg/mL salmon sperm DNA. Samples were further incubated for 16 h at 42 °C with fresh hybridisation buffer supplemented with 0.2 ng/µL heat-denatured digoxigenin-labelled ALU probe, which was synthesised by PCR as described previously [19]. Detection was performed using anti-Digoxigenin alkaline phosphatase-conjugated Fab fragments (Roche Diagnostics) and NBT/BCIP (Linaris) as substrate. Sections were counterstained with Methyl Green (Linaris), mounted with Neomount (Merck-Millipore, Burlington, MA, USA) and imaged using a Keyence BZ-X800 microscope (Keyence).

### 2.12. Immunohistochemistry

Formalin-fixed, paraffin-embedded tissue sections were deparaffinised in Roti–Histol (Carl Roth) and rehydrated with isopropanol. Antigen retrieval was performed using Dako target retrieval buffer pH 6 (Dako, Hamburg, Germany) in a pressure cooker at 121 °C for 5 min. Sections were blocked with PBS supplemented with 5% BSA and incubated with the primary antibody specific for cleaved caspase-3 (Cell Signaling, Leiden, The Netherlands) diluted 1:250 in PBS/1% BSA overnight at 4 °C. Detection was carried out using the BrightVision plus kit (VWR International, Bruchsal, Germany) according to the manufacturer’s instructions. ImmPACT Vector Red (Linaris) was used as substrate. Samples were counterstained with Gill´s haematoxilin (Santa Cruz) and mounted with NeoMount (VWR International).

### 2.13. Statistics

Descriptive statistics, such as mean and standard deviation, were calculated using SPSS software (Version 25; IBM, Armonk, NY, USA). Since group sizes vary between *n* = 6 and *n* = 20 in case of the CAM assays, normal distribution of the data cannot be assumed in all cases. We therefore used the non-parametric Mann–Whitney U-test to compare experimental groups, with *p*-values < 0.05 regarded as statistically significant, and *p* < 0.01 as highly significant. Data are presented as mean ± SD. The given number of n always refers to the number of biological replicates (cell lines or eggs in CAM assays).

## 3. Results

First, we aimed to test whether and to which extend OS cell lines are sensitive to the proposed drug combination consisting of the two MCI inhibitors: Metformin and Fenofibrate, and the H^+^-ionophore Monensin (MeMoFe). This drug combination is expected to increase the rate of glycolysis and lactate production by blocking the MCI, leading to intracellular acidification resulting in ATP depletion due to the H^+^-ionophore-mediated inhibition of the pHi compensation. Thus, these drugs comprise the aforementioned “Warburg Trap” and are therefore referred to as “Warburg-Trap drugs” (WT drugs) in the following.

**Figure 1:**
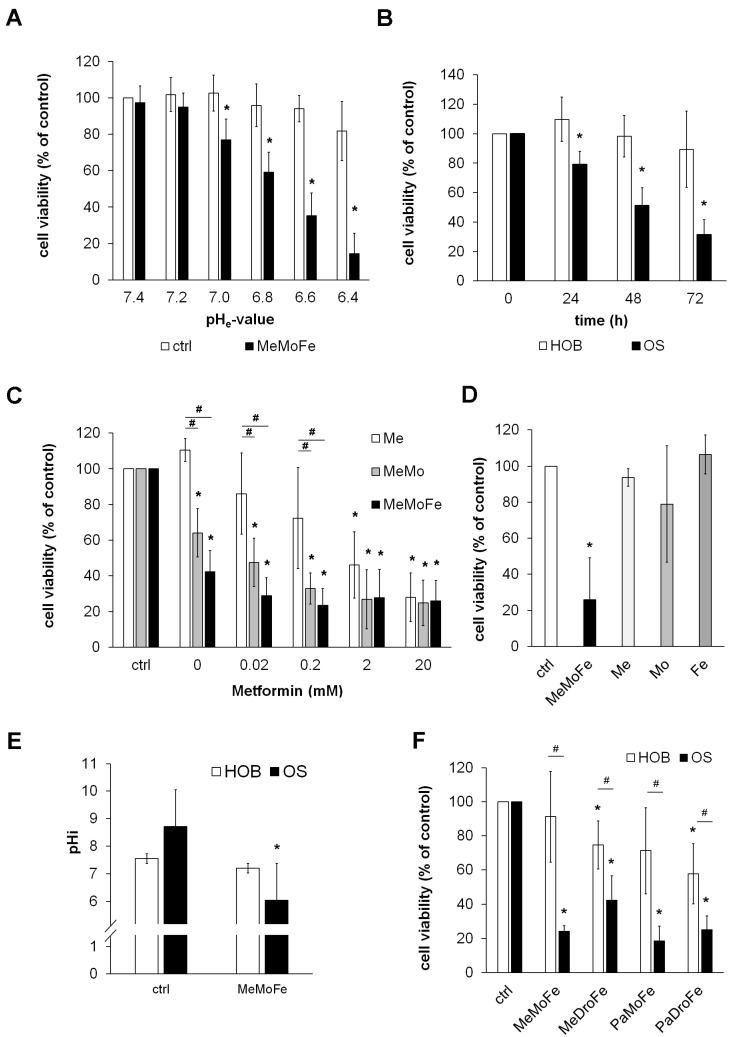
Cytotoxic effects of Warburg-Trap drugs on cancer cells. (**A**) Six OS cell lines (*n* = 6) were treated with a combination of Metformin (1 mM), Monensin (5 nM) and Fenofibrate (10 µM) (MeMoFe) in culture media with pH values ranging from 6.4 to 7.4. Cell viability was quantified after 72 h by WST-1 assay and is expressed as percent of untreated cells cultured at pH 7.4 (* *p* < 0.01 compared to untreated cells). (**B**) OS and HOB cell lines (*n* = 6 each) were treated with a combination of Metformin (1 mM), Monensin (5 nM) and Fenofibrate (10 µM) in an acidic culture medium (pH 6.5). Cell viability was quantified at the indicated time points by WST-1 assay and is expressed as percent of untreated control cells (* *p* < 0.01 compared to untreated control cells). (**C**) OS cell lines (*n* = 6) were treated with Metformin (Me) in the indicated concentrations with or without the addition of Monensin (5 nM) (MeMo) or a combination of Monensin (5 nM) and Fenofibrate (10 µM) (MeMoFe). Cell viability was quantified after 72 h by WST-1 assay and is expressed as percent of untreated control cells (* *p* < 0.01 compared to untreated control cells, # *p* < 0.01 compared to cells treated with metformin alone). (**D**) OS cell lines (*n* = 6) were treated with a combination of Metformin (1 mM), Monensin (5 nM) and Fenofibrate (10 µM) (MeMoFe) as well as all three drugs alone at the same concentrations. Cell viability was quantified after 72 h by WST-1 assay and is expressed as percent of untreated control cells (* *p* < 0.01 compared to untreated control cells). (**E**) WT drugs induce intracellular acidification in OS cells. OS and HOB cell lines (*n* = 6 each) were cultured for 24 h in an acidic medium (pH 6.5) with or without the addition of Metformin (1 mM), Monensin (5 nM) and Fenofibrate (10 µM) (MeMoFe) before pHi was quantified (* *p* < 0.01 compared to untreated control cells). (**F**) Viability of OS and HOB cells after treatment with different WT drug combinations. OS and HOB cells (*n* = 6 each) were treated with different combinations of Metformin (1 mM), Monensin (5 nM), Fenofibrate (10 µM), Drotaverine (10µM), and Papaverine (10 µM) in acidic culture medium (pH 6.5). Cell viability was quantified after 72 h by WST-1 assay and is expressed as percent of untreated control cells. (* *p* < 0.05 compared to untreated cells, # *p* < 0.05 OS compared to HOB cells).

The proposed mechanism strongly depends on a gradient between extracellular pH value (pH_e_) and intracellular pH value (pH_i_). Therefore, the cytotoxic effects of the WT drugs were tested on six osteosarcoma cell lines cultured in media with different pH values. We observed that the cytotoxic effect was strongly dependent on the pH_e_. While the WT drugs did not affect cell viability when used at physiologic pH_e_ 7.4, cell viability dropped below 20% after 72 h treatment at pH_e_ 6.4 (Figure 1A). Notably, the effective pH range was comparable to the one found in the microenvironment of most tumours ranging from 6.5 to 7.0 [22]. Since this acidification is based on increased glucose metabolism and lactate production, which is a characteristic feature of cancer cells, we hypothesised that non-neoplastic cells like primary human osteoblasts (HOBs) will remain unaffected. Indeed, the viability of HOBs was not significantly reduced by drug treatment for up to 72 h even in an acidic environment (Figure 1B). Many studies already reported anti-cancer properties of Metformin; however, to significantly reduce tumour growth and proliferation, very high Metformin concentrations were necessary. When applied as a single drug agent, a concentration of 20 mM was necessary to reduce cell viability to 28% after 72 h treatment. In contrast, 0.2 mM Metformin was sufficient to reduce cell viability to 33% when combined with Monensin and to 24% when combined with both Monensin and Fenofibrate. Moreover, a Metformin concentration of 0.02 mM in the drug combination was sufficient to achieve an effect comparable to 20 mM Metformin alone. These data indicate that sensitivity of cancer cells toward Metformin in the WT drug combination is 100–1000-fold higher compared to Metformin alone (Figure 1C). As expected, Monensin and Fenofibrate at the same concentrations as used in the WT drug combination did not induce any significant changes in cell viability when used as a single agent (Figure 1D).

Next, we analysed the impact of the WT drug treatment on intracellular pH values (pH_i_). OS and HOB cells were kept in a culture medium with a pH value of 6.5 to stimulate the acidic tumour microenvironment with or without the addition of WT drugs. After 24 h, the pH_i_ was analysed using the fluorescent pH-indicator BCFL. To generate standard curves, cells were kept in a culture medium with adjusted pH values ranging from 6.0 to 8.0 (pH_e_). To ensure that pH_i_ values will adjust to the pH_e_ values, Nigericin was added to the culture medium. Nigericin acts as a potassium ionophore disrupting the intracellular H^+^ and K^+^ concentrations, thus triggering an adaptation of the pH_i_ to the pH_e_. Using this approach, we detected alkaline pH_i_ values in untreated OS cells ranging from 8.1 to 9.3. Treatment with the WT drugs significantly reduced pH_i_ to values ranging from 5.6 to 6.7. In contrast, the mean pH_i_ of HOBs cultured under the same conditions in an acidic culture medium was 7.5 and showed no significant change after treatment with the WT drugs (Figure 1E). The pHi values of the individual cell lines and the corresponding standard curves are shown in the Supplementary Figure S1. From these data, we conclude that in contrast to non-cancer HOBs, all tested OS lines show a dramatic drop in pHi upon treatment with WT drugs.

Since the Warburg Trap approach is based on a combination of MCI inhibitors and H^+^-ionophores, we next analysed the effects of further drug combinations using the MCI inhibitors Metformin, Fenofibrate, or Papaverine, either with the ionophore Monensin or with Drotaverine, an anti-spasmodic drug that also causes a pHi drop and regulates cAMP and calcium levels. We aimed to test whether the ionophore Monensin, a drug that is widely used in ruminant animal feeds but still has not been FDA approved for human use, can be substituted with Drotaverine that is FDA approved, safe, and shows a good DMPK (drug metabolism and pharmacokinetics) profile in humans [23]. All tested drug combinations caused a significant reduction in OS cell viability. While the initially investigated drug combination of Metformin, Monensin and Fenofibrate (MeMoFe) reduced cell viability after 72 h treatment to 24.4% compared to untreated cells, replacement of Monensin by Drotaverine (MeDroFe), replacement of Metformin by Papaverine (PaMoFe), or replacement of Metformin and Monensin with Papaverine and Drotaverine (PaDroFe) reduced cell viability to 42.5, 18.7, and 25.2%, respectively. In contrast, the viability of HOBs was significantly less affected (Figure 1F). We conclude that the Warburg trap is not dependent on specific medications but can rather be achieved with various drug combinations, including FDA-approved ones.

**Figure 2:**
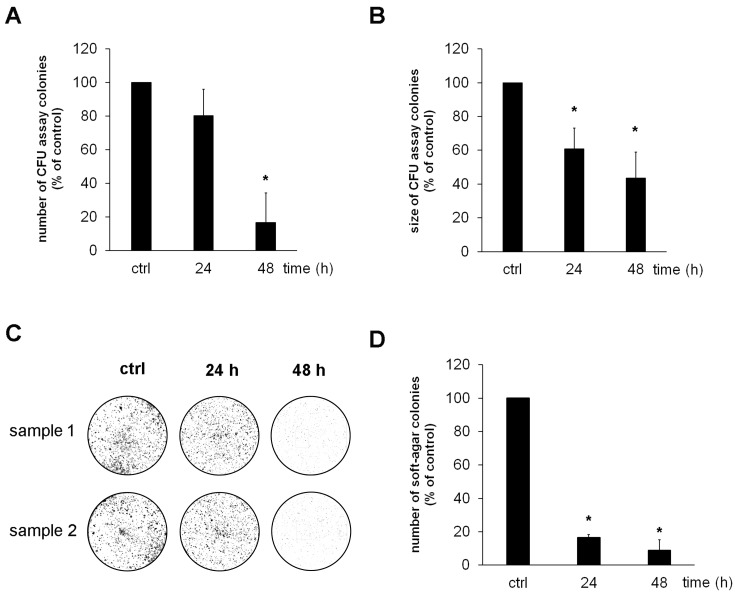
Warburg Trap drugs significantly reduce the colony-forming capacity of OS cells. OS cell lines (*n* = 6) were seeded at low density in culture plates and treated with the WT drugs (Metformin (1 mM), Monensin (5 nM), and Fenofibrate (10 µM)) in an acidic culture medium (pH 6.5). After 24 h and 48 h of treatment, the culture medium was replaced by a normal cell culture medium with a physiological pH value of 7.4. Colonies that formed during a further seven days of culture were stained with haematoxylin and photographed before (**A**) the number of colonies and (**B**) the size of the colonies were quantified using the software ImageJ version 1.48v (* *p* < 0.01 compared to untreated controls). (**C**) Representative photographs of stained colonies obtained with cell line 143B analysed in duplicates. (**D**) Analysis of colony numbers that formed after pre-treatment of osteosarcoma cell lines (*n* = 6) with WT drugs and subsequent cultivation for 14 days in soft agar (* *p* < 0.01 compared to untreated controls).

To evaluate the influence of the WT drugs on tumour cell growth and proliferation, we used colony-forming unit (CFU) and soft-agar assays. The CFU assays were used to identify cells with cancer stem cell (CSCs)-like properties by analysing the capability of single cells to form colonies through clonal expansion. The soft-agar assays were used to monitor the ability of cells to grow anchorage-independent in semi-solid matrices, which is a key feature of transformed tumour cells. Treatment of osteosarcoma cells with WT drugs significantly reduced the number and size of colonies that formed in CFU assays (Figure 2A–C). WT drug treatment further significantly reduced the number of colonies that formed in soft agar (Figure 2D). As expected, and in contrast to OS cells, HOBs did not form any colonies in CFU and soft agar assays.

**Figure 3:**
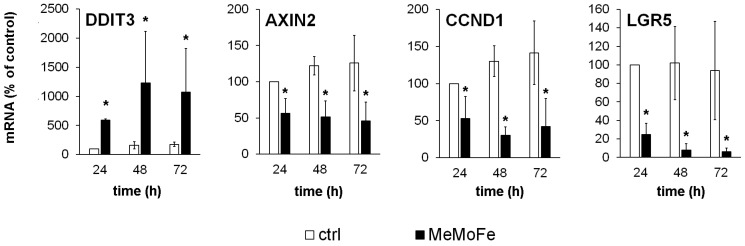
*DDIT3*-mediated downregulation of WNT target genes by WT drugs. Osteosarcoma cell lines (*n* = 3) were treated with WT drugs (Metformin (1 mM), Monensin (5 nM), and Fenofibrate (10 µM)) (MeMoFe) for the indicated time before the expression of *DDIT3* and the WNT target genes *AXIN2*, *CCND1* and *LGR5* were analysed by real-time quantitative RT-PCR analysis. The expression of the reference gene *RPS13* (ribosomal protein S13) was used for normalisation. Data are presented as percent of untreated 24 h controls (* *p* < 0.05 compared to untreated controls).

Earlier, we demonstrated that the WT drugs induce intracellular acidification via mechanisms involving cancer-specific inhibition of the WNT signalling pathway. We showed that this inhibition is mediated by the global transcriptional repressor DDIT3 (DNA damage-inducible transcript 3), which is known to be expressed in response to a wide variety of cellular stresses [14]. In this study, we confirmed a strong WT drug-induced DDIT3 upregulation also in osteosarcoma cells. As a consequence, we observed a significant downregulation of several WNT target genes, including canonical WNT marker genes: AXIN2 (axis inhibition protein 2), CCND1, encoding the cell cycle regulator cyclin D1, and LGR5 (G-protein coupled receptor 5) that is also known as a bona fide cancer stem cell marker [24] (Figure 3).

**Figure 4:**
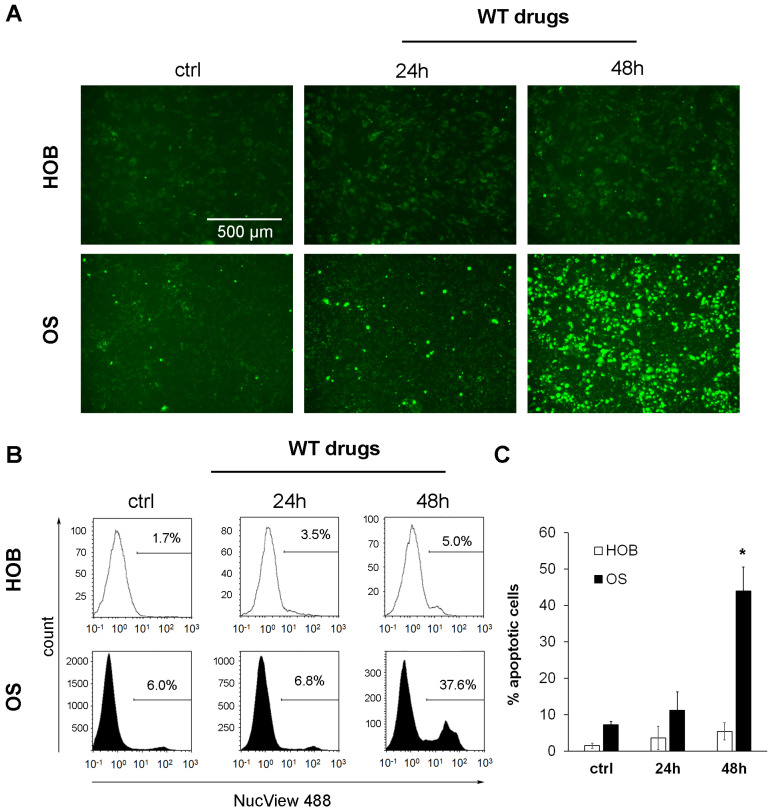
WT drugs induce apoptosis in OS cells but not in HOBs. OS and HOB cell lines (*n* = 3 each) were treated with the WT drugs Metformin (1 mM), Monensin (5 nM) and Fenofibrate (10 µM) for 24 and 48 h in an acidic culture medium (pH 6.5). Control cells were cultured in an acidic medium without the addition of WT drugs. After treatment, apoptotic cells were stained with NucView 488. (**A**) Representative fluorescence images of NucView 488-positive HOB and OS cells (green) at a magnification of 100×. (**B**) Representative flow cytometry analyses of apoptotic HOB and OS cells. (**C**) Quantification of apoptotic HOB and OS cells (*n* = 3 each) (* *p* < 0.01 compared to untreated controls.

From these data, we conclude that WNT signalling, important for cancer stem cell survival, is repressed already after 24 h of treatment. Since the loss of WNT signalling in cancer stem cells is supposed to induce apoptosis, we next addressed the question of whether the WT drug-induced cell death is also mediated by apoptotic mechanisms using NucView 488 staining. Upon cleavage by active caspase-3, a hallmark of apoptosis, NucView 488 migrates to the nucleus and stains DNA with bright green fluorescence. Using fluorescence microscopy and flow cytometry, we were able to detect a strong induction of apoptosis in WT drug-treated OS cells, which was absent in HOBs (Figure 4A–C).

**Figure 5:**
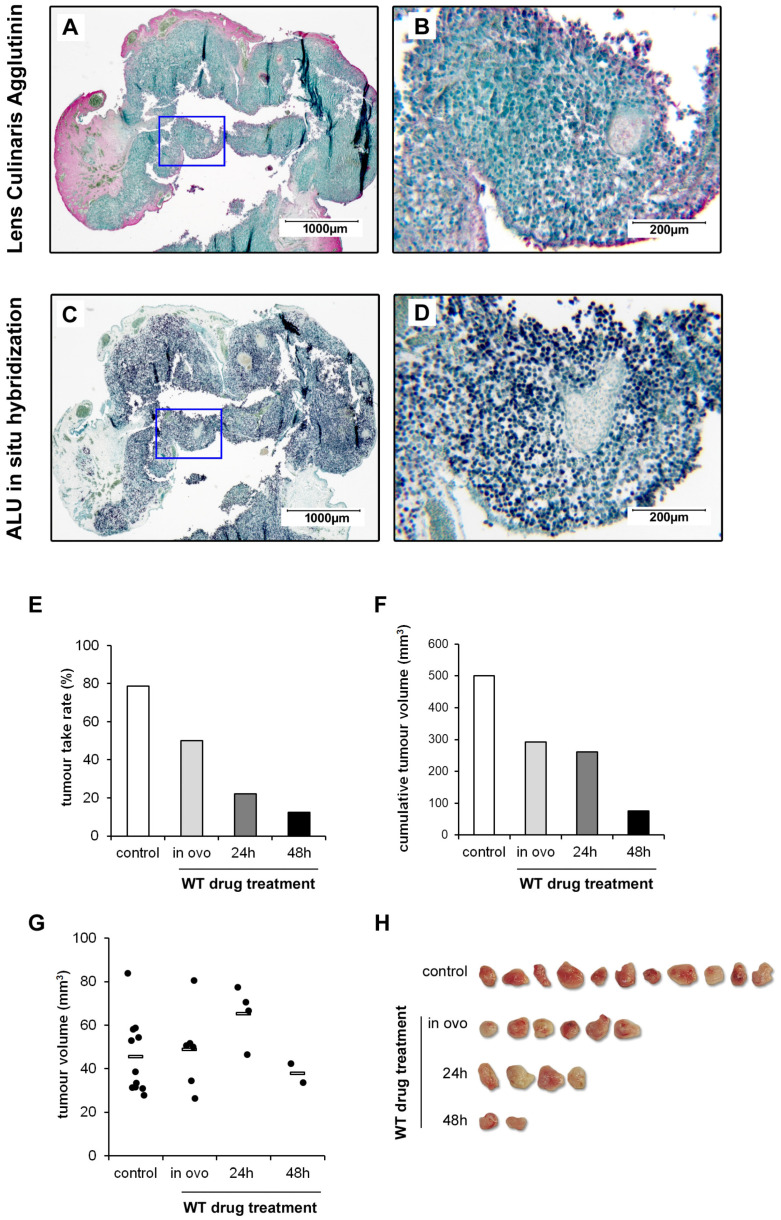
WT drugs inhibit tumour formation in vivo. Characterisation of the resected tumour xenografts was made using (**A**,**B**) Lens Culinaris Agglutinin staining, showing the unstained tumour tissue surrounded by the red-stained CAM and (**C**,**D**) hybridisation of the xenograft with a probe specific for the human ALU DNA sequence (black) detecting the human 143B OS cells. The blue frames define the inserts shown in a higher magnification on the right. All samples were counterstained with Methyl Green. For WT drug treatment, 143B cells were either pre-treated with the drugs Metformin (1 mM), Monensin (5 nM) and Fenofibrate (10 µM) (MeMoFe) for 24 h and 48 h or the drugs were applied in ovo together with the tumour cells (1 × 10^6^ cells per egg) directly onto the CAM of fertilised chicken eggs (*n* ≥ 20 per experimental group). After seven days, tumours that have formed were resected and (**E**) the tumour take rate, (**F**) the cumulative tumour volumes and (**G**) the individual tumour volumes were calculated. (**H**) Images of the resected tumours. Data from a representative experiment are shown.

The impact of the Warburg-Trap approach on tumour formation and growth under in vivo conditions was analysed using a chicken chorioallantoic membrane (CAM) assay that we have previously established and optimised for the xenotransplantation of osteosarcoma cells [19]. The transplantation of OS cells (143B) onto the CAM of fertilised chicken eggs resulted in the formation of solid xenografts within seven days. The human origin of the tumour-forming cells was verified by in situ hybridisation with a human *ALU* probe and differentiated from chicken cells by a Lens Culinaris Agglutinin staining (Figure 5A–D).

To analyse the effects of Warburg Trap drugs on tumour formation, the drugs were either directly delivered in ovo together with the cells, or cells were pre-incubated with the WT drugs in vitro before transplantation of the remaining viable cells.

Treated and non-treated cells were transplanted onto the CAM of chicken eggs (*n* = 20 eggs each group) on day 9 of the embryonal development. Eggs were further incubated for 7 days before tumours that have formed were resected. Eggs with dead embryos and contaminated eggs were excluded from the experiment. Tumour take rates, indicating the percentage of eggs with tumour development and the volumes of the individual tumours were calculated. Both in ovo treatment and pre-treatment of the cells for 24 h or 48 h reduced the tumour take rates to 50%, 22% and 13% in comparison to 79% observed with untreated control cells (Figure 5E). Moreover, the cumulative tumour volumes decreased from 501 mm^3^ to 292 mm^3^, 260 mm^3^, and 76 mm^3^, respectively (Figure 5F). In contrast to the number of tumours, the individual tumour size was not significantly affected by the drug treatment (Figure 5G,H).

**Figure 6:**
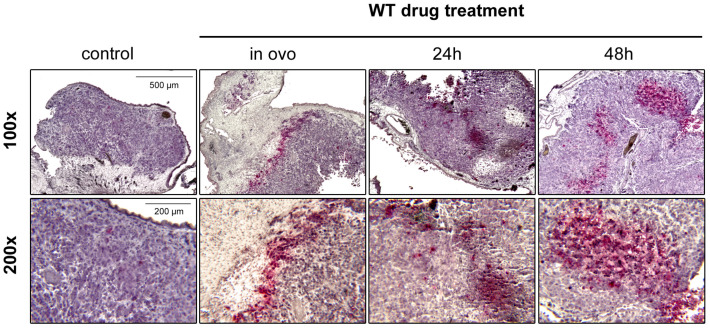
WT drugs induce apoptosis in vivo. Tumours that formed after pre-treatment of OS cells (HOS 143B) with the drug combination Metformin (1 mM), Monensin (5 nM) and Fenofibrate (10 µM) for 24 h and 48 h or treated directly in ovo were stained for cleaved caspase-3 (red), counterstained with haematoxilin (violet) and photographed at 100× and 200× magnification. Representative photographs are shown.

Induction of apoptosis by WT drugs in vivo was performed by immunohistochemical analysis of cleaved caspase-3 in the resected tumour xenografts. In contrast to the untreated controls, tumours that have been treated in ovo and those that developed from pre-treated cells showed regions with considerable amounts of cleaved caspase-3 positive and thus apoptotic cells (Figure 6).

## 4. Discussion

While OS patients without recurrent or metastatic disease dramatically benefited from the introduction of chemotherapy in the late 1970s, with an increase in the 5-year overall survival rate from <20% up to 60–70% [25], the overall survival rate for OS patients with a recurrent or metastatic disease remains very poor [3,4]. Unfortunately, despite the development of modified treatment protocols and in contrast to most other solid tumours, outcomes for OS patients could not be further improved over the past decades, especially for metastatic diseases. In addition, the development of chemoresistance and the occurrence of severe side effects in response to high-dose chemotherapy which are often observed in the course of OS therapy [26] further emphasise an urgent need for a new therapeutic strategy to support OS patients.

The beneficial effects of Metformin on tumour prevention have already been extensively studied [27,28,29]. For a long time, Metformin has been regarded as a universal but weak anticancer drug, although the exact mechanisms underlying these effects were not fully understood. In our previous work, we deciphered an exact mode of action for Metformin, and based on this mechanism, we proposed a new tumour therapy approach that exploits the distinct features of cancer cell metabolism and named it “Warburg Trap”. We were able to prove that Metformin can trigger a pHi drop in vitro and in vivo and in addition to that acts as a WNT signalling inhibitor. We speculated that these effects underly the known universal anti-cancer effects of Metformin. We could further demonstrate that Metformin inhibits the WNT signalling pathway via intracellular acidification, induction of DDIT3 and subsequent disruption of the TCF4/β-catenin activation complex. Since the anticancer effects of Metformin alone are very limited, we further suspected that a combination of Metformin with ionophores would trigger an auto-enhancing cycle that finally leads to cell death [14]. In the current study, we hypothesised that osteosarcoma cells would also be sensitive to this kind of treatment that utilises drugs at nM or low µM concentrations; thus, it is less toxic for normal non-cancer cells.

Although WT drug-induced cell death is primarily the result of intracellular acidification and the inability of cancer cells to restore physiological pHi, the observed inhibition of the WNT signalling pathway may additionally contribute to the observed cell death. In this respect, the known aberrant WNT signalling in OS cells [30] further supports our hypothesis of a possible treatment option for OS by WT drugs. The aberrant activation of WNT signalling has been implicated in the development of osteosarcoma and, as a consequence, the suppression of WNT/β-catenin-mediated transcription has been shown to inhibit the proliferation, migration, invasion, and colony formation of OS cells [30]. WNT signalling has further been shown to regulate the early and late stages of apoptosis [31]; thus, the WT drug-mediated inhibition of WNT signalling might additionally contribute to the observed induction of apoptosis in OS cells. A possible mechanism might involve inhibition of Wnt-1, which has been shown to inhibit apoptosis by preventing the release of cytochrome C from mitochondria and to inhibit the activity of caspase-9, which is a key player within the intrinsic apoptosis pathway [32]. Further, a significant downregulation of the apoptosis inhibitor survivin (BIRC5) that has already been observed in OS cells after treatment with an WNT-/β-catenin inhibitor might also contribute to the WT drug-induced cell death [30].

In addition to the aberrant WNT signalling in OS cells, the fact that a reversal of the pH gradient has already been shown to reduce multidrug resistance caused by Doxorubicin in OS cells further implies that osteosarcoma cells are sensitive to the WT drug treatment [33]. In our previous work, we concluded two main triple-drug combinations that demonstrated the most versatile effects in terms of very strong and targeted action in cancer cells with no or very minor cytotoxic side effects in non-cancer cells (patent WO2020/234454A1; Deutsches Krebsforschungszentrum; Combination treatment of cancer targeting energy metabolism and intracellular ph; 26 11 2020).

To establish the Warburg Trap in OS cells, we investigated several drug combinations consisting of the MCI inhibitors Metformin, Fenofibrate, or Papaverine, with the ionophores Monensin, or Drotaverine (also known for its ability to cause a drop of the intracellular pH value). Metformin, the diabetes type II prescription drug, is well tolerated by patients, even when consumed over several years and decades. Fenofibrate is a class of lipid-lowering medication with properties of both MCIi and Monocarboxylate transporter 4 inhibitors (MCT4is), which is prescribed to treat abnormal blood lipid levels. Both drugs have been approved by pharmacopoeia for human use. Monensin is an ionophore that is currently widely used in veterinary and ruminant animal feeds as an anticoccidial drug. Unfortunately, it has not been FDA approved for use in human patients yet. Nevertheless, it features in several clinical trials as an antibiotic for external application to treat skin ulcer wounds. It has been shown to display no toxic effects in many different animals up to a plasma concentration of 41 µM [34], which is significantly lower compared to the concentrations applied in our in vitro (5 nM) and in vivo studies (100 µg/mg PLGA formulation that is equivalent to 1.5 nmol/injection/animal) (patent WO2020/234454A1). Even though no data on Monensin intake in humans are available to date, no adverse effects were observed in animals treated with Monensin alone or with Monensin applied in a drug combination over a prolonged period with a daily uptake (up to 1 year) in our previous in vivo experiments. These experiments were based on a mouse xenograft tumour model induced by DLDL1 colon adenocarcinoma cells as well as a spontaneous mammary gland cancer C3(1) mouse model [35] (data not shown).

In addition to the analysis of the WT drug combination Metformin, Monensin and Fenofibrate, we investigated whether individual drugs might be substituted without any reduction in the cytotoxic effect and tumour cell specificity. We analysed the replacement of Monensin by Drotaverine as well as the replacement of Metformin by Papaverine. Drotaverine (commercial name “No-Spa”) is an FDA-approved, anti-spasmodic drug that seems to regulate cAMP and calcium balance and might thus be able to replace the ionophore Monensin. Drotaverine is not toxic and can be prescribed even to pregnant women. The anti-spasmodic drug Papaverine is also FDA approved and is used to treat many conditions that cause spasms of smooth muscles. Since inhibition of the MCI by Papaverine has already been shown, we assumed that Papaverine might be another substitute for Metformin in the WT drug mix. The analysis of the cytotoxic effects of these different drug combinations revealed that the Warburg Trap effect is not restricted to specific drugs but can be achieved by several drug combinations as long as MCI inhibitor and ionophore activities synergise. These data demonstrate that the Warburg Trap may be established exclusively with FDA-approved drugs, allowing a rapid transfer of our results into the clinic.

Interestingly, in addition to influences on cell cycle progression, proliferation, and induction of autophagy and apoptosis, a selective targeting of CSCs by Metformin has already been demonstrated [36]. According to the cancer stem cell hypothesis, this self-renewing subpopulation within the tumour displays a chemoresistant phenotype capable of regenerating the tumour tissue after chemotherapy, thus leading to tumour relapse. A combination of different chemotherapeutic agents with Metformin has been shown to inhibit tumour growth and relapse of mouse breast cancer xenografts, while the chemotherapeutic drugs alone could not prevent relapse. Furthermore, in combination with Metformin, the effective dose of Doxorubicin for the prevention of tumour relapse could be reduced 4-fold [37]. These data indicate that Metformin may contribute to a reduction in the required dose of cytotoxic drugs and thus the extent of unwanted negative side effects during chemotherapy. In this context, our observation that WT drugs target CSC also in OS suggests that a WT drug-based treatment represents a promising approach to support current OS chemotherapy protocols.

## 5. Conclusions

We demonstrated that the WT drug combination Metformin, Fenofibrate and Monensin and further drug combinations based on MCI inhibitors and H+ ionophores induce intracellular acidification in OS cells with subsequent induction of an apoptotic cell death that could not be observed in non-neoplastic human osteoblasts. As a consequence, tumour formation in vivo was significantly reduced. The identified tumour cell specificity together with the observation that CSCs are also targeted by WT drugs suggests that the Warburg Trap is a promising mechanism for the development of new therapeutic strategies for the treatment of osteosarcoma and/or to support conventional chemotherapy with the aim of preventing tumour relapse. The fact that an effective WT drug combination might be based exclusively on FDA-approved substances can facilitate the transfer of this novel approach into the clinic.

## Data Availability

The data generated in the presented study are available within the article and its supplementary data files. Materials are available from the corresponding author on upon request.

## Supplementary Materials

The following supporting information can be downloaded at https://www.mdpi.com/article/10.3390/cells13010061/s1, Table S1: Primer used for RT-qPCR analyses; Figure S1: Quantification of pHi in OS and HOB cell lines.
