# Supplementary material for: The Warburg Trap: A Novel Therapeutic Approach for Targeting Osteosarcoma
Source: Cells. 2023 Dec 27;13(1):61. doi: 10.3390/cells13010061 (PMC10778102; doi:10.3390/cells13010061)
Supplement: Supplementary file 1 [file cells-13-00061-s001.zip › Supplementary Figure S1.pdf]

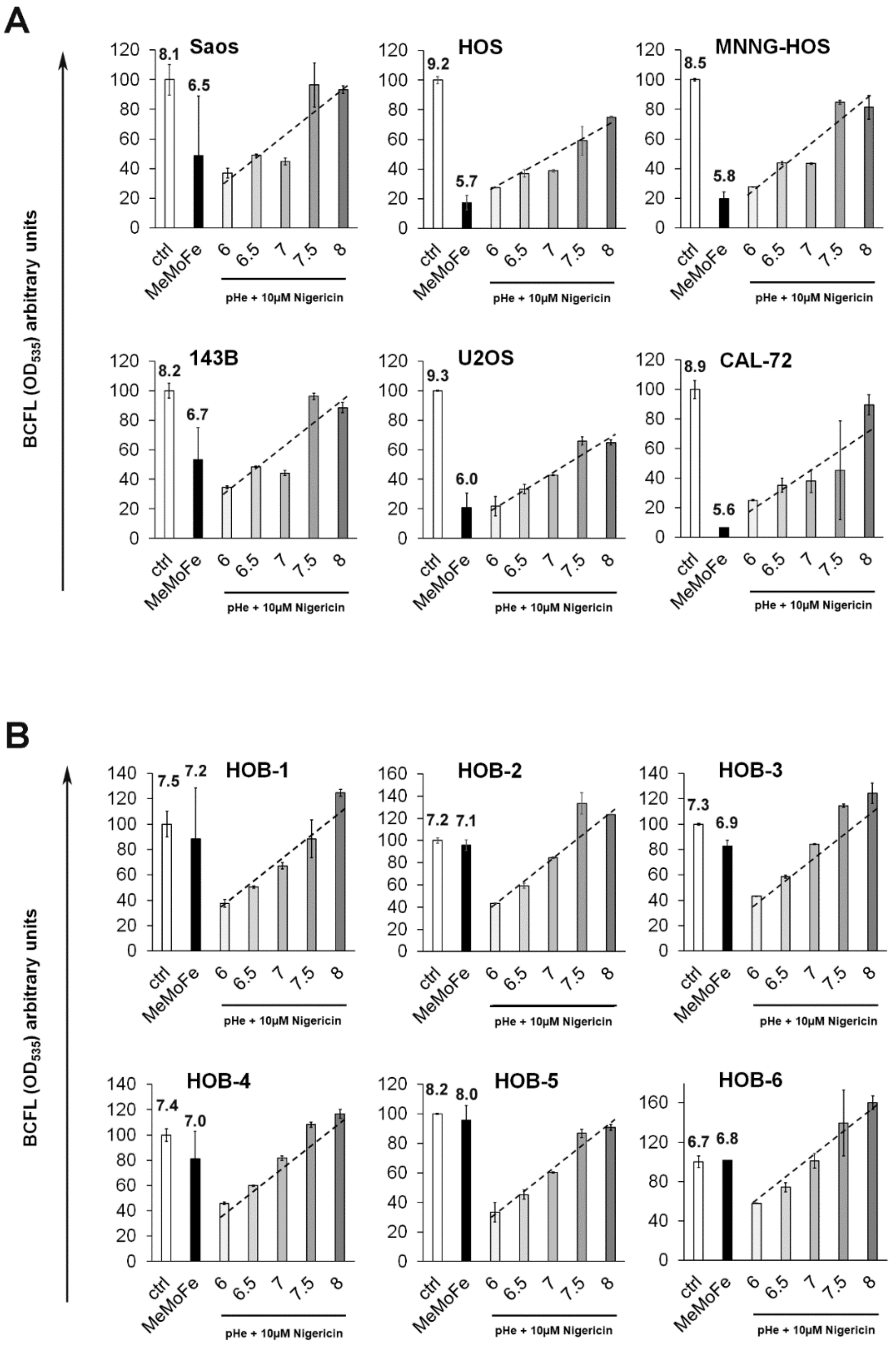


**Supplementary Figure S1.** Quantification of pHi in OS and HOB cell lines (n=6 each). **A)** OS cells and **B)** HOBs were cultured for 24 h in acidic medium (pH 6.5) with or without the addition of Metformin (1 mM), Monensin (5 nM) and Fenofibrate (10 µM) (MeMoFe). Standards were generated using culture medium with the indicated pH-values ranging from 6.0 to 8.0 and the addition of the ionophore Nigericin (10 µM). After labelling of the cells with the pH-indicator BCFL, fluorescence was quantified in a fluorometer. Calculated pHi values are indicated.
