# Supplementary material for: The Warburg Trap: A Novel Therapeutic Approach for Targeting Osteosarcoma
Source: Cells. 2023 Dec 27;13(1):61. doi: 10.3390/cells13010061 (PMC10778102; doi:10.3390/cells13010061)
Supplement: Supplementary file 1 [file cells-13-00061-s001.zip › Supplementary Table S1.pdf]

Supplementary Table S1. Primer used for RT-qPCR analyses

Gene	Forward primer (5´- 3´)	Reverse primer (5´- 3´)
RPS13	GGTTGAAGTTGACATCTGACGA	CTTGTGCAACACCATGTGAAT
DDIT3	CAGAGCTGGAACCTGAGGAG	TGGATCAGTCTGGAAAAGCA
AXIN2	CCACACCCTTCTCCAATCC	TGCCAGTTTCTTTGGCTCTT
CCND1	CAGATCATCCGCAAACACGC	AAGTTGTTGGGGCTCCTCAG
LGR5	ACCAGACTATGCCTTTGGAAAC	TTCCCAGGGAGTGGATTCTAT
